# Research on the multitarget 3D trajectory tracking method of *Thalassodes immissaria* in the thermal infrared region based on YOLOX-GMM and SORT-Pest

**DOI:** 10.3389/fpls.2024.1403421

**Published:** 2024-10-07

**Authors:** Xinrui Qiu, Juan Xia, Ye Zeng, Guangwen Huang, Bolai Xin, Runpeng Jiang, Kaixuan Wu, Zhe Ma, Jun Li

**Affiliations:** ^1^ College of Engineering, South China Agricultural University, Guangzhou, China; ^2^ Guangdong Laboratory for Lingnan Modern Agriculture, Guangzhou, China; ^3^ State Key Laboratory of Agricultural Equipment Technology, Beijing, China

**Keywords:** *Thalassodes immissaria*, thermal infrared, 3D trajectory, target tracking, background modeling

## Abstract

**Introduction:**

Studying the behavioral responses and movement trajectories of insects under different stimuli is crucial for developing more effective biological control measures. Therefore, accurately obtaining the movement trajectories and behavioral parameters of insects in three-dimensional space is essential.

**Methods:**

This study used the litchi pest *Thalassodes immissaria* as the research object. A special binocular vision observation system was designed for nighttime movement. A thermal infrared camera was used for video recording of *T. immissaria* in a lightless environment. Moreover, a multi-object tracking method based on the YOLOX-GMM and SORT-Pest algorithms was proposed for tracking *T. immissaria* in thermal infrared images. By obtaining the central coordinates of the two *T. immissaria* in the video, target matching and 3D trajectory reconstruction in the parallel binocular system were achieved.

**Results:**

Error analysis of the *T. immissaria* detection and tracking model, as well as the 3D reconstruction model, showed that the average accuracy of *T. immissaria* detection reached 89.6%, tracking accuracy was 96.9%, and the average error of the reconstructed 3D spatial coordinates was 15 mm.

**Discussion:**

These results indicate that the method can accurately obtain the 3D trajectory and motion parameters of *T. immissaria*. Such data can greatly contribute to researchers' comprehensive understanding of insect behavioral patterns and habits, providing important support for more targeted control strategies.

## 1 Introduction

Insect behavior research has significant importance in plant protection, especially with the rapid development of agriculture, and the importance of insect behavior analysis has become increasingly prominent (Mo et al., 2006). Researchers mainly develop scientific and rational pest control measures by observing the behavioral responses of insects to different stimuli, including movement trajectories, foraging frequency, and mating. This approach aims to reduce pesticide use and improve the natural environment (Ke et al., 2021; Cheng et al., 2021). Among these, the information on insect movement trajectories is intuitive and rich, can directly express the insect’s tendencies, and can be combined with the experimental environment as an important basis for studying their social behaviors such as mating and foraging. while Dong et al. (2020) analyzed the color preference behavior of stored-grain pests based on their movement trajectories.

In the past, insect behavior analysis was typically performed manually, requiring professionals to observe and record insect behavior in the observation box; however, this method has problems such as qualitative and arbitrary judgements and time-consuming processes (Wu et al., 2009). With the development of computer vision technology, researchers have gradually applied it to insect behavior analysis. For example, Gaydecki (1984) first achieved moth trajectory tracking and behavior analysis under field conditions by setting up mercury lamps to attract night moths, using cameras to capture moth motion videos, and finally obtaining trajectories by manually marking the moths in the video. Although this method does not require constant observation by researchers, it still requires frame-by-frame video analysis, which is a considerable workload.

With the continuous development of computer hardware and software, image processing technology has made great progress. Guo et al. (2018) used KCF filters to model and match targets, tracking the diamondback moth and 3D trajectory reconstruction through two orthogonal RGB cameras. Although this method can accurately obtain the 3D trajectory of the diamondback moth, it can only track a single target and requires manual selection of the tracking target in the first frame, with low automation. There are also some studies that use harmonic radar (Riley et al., 1996), radio frequency identification (Klein et al., 2019), and acoustic sensors (Heise et al., 2017) to track insects; however, most of these methods require manual data processing, which has low efficiency and accuracy.

Deep learning methods provide new directions for the development of computer vision (Christin et al., 2019). Because of their excellent ability to learn complex image features, an increasing number of researchers have begun to use deep learning technology to process collected image data to complete various tasks (Wang et al., 2019; Lv et al., 2023; Zhang X. Y. et al., 2023). In insect behavior analysis, Ngo et al. (2021) successfully tracked the trajectory of bees using deep learning and judged whether they carried pollen. Nasir et al. (2021) achieved bee recognition and 3D trajectory acquisition by fusing RGB cameras with depth cameras. Hong et al. (2022) proposed a multiobject tracking method based on deep learning and spatiotemporal features for classifying fruit fly behaviors. However, these methods usually require additional lighting, and the quality of target tracking is often affected by lighting. Additionally, many insects are prone to night activity, and even weak light can affect their behavior, leading to biased behavioral analysis (Tu et al., 2014).

Based on existing research on insect trajectory tracking, problems such as small insect targets that are difficult to detect, fast and irregular insect flight, and insufficient research on the detection and 3D trajectory reconstruction of insects under no lighting conditions with low accuracy exist.

With the reduction in the cost of thermal infrared cameras, they have gradually become effective tools in computer vision because they can image objects regardless of lighting conditions (Liang and Li, 2023; Liu et al., 2024; Lang et al., 2023). However, thermal infrared images have low image resolution and lack color texture, making feature extraction more difficult and resulting in high false positives and missed detections (Zhang R. et al., 2023). Therefore, few people have applied thermal infrared cameras to the study of insect behavior trajectories.

*T. immissaria* is a major pest in litchi and longan orchards. It hides among the leaves during the day and becomes active only at night. It is characterized by rapid reproduction and ovulation, feeds on the tips of fruit trees, posing a considerable threat to the stable production of litchi and longan (Chen et al., 2010, 2017). Traditional control measures involving pesticide spraying raise concerns about food safety, and due to increasingly strict restrictions on insecticide use, such pest control methods will gradually be phased out (Dias et al., 2018). In recent years, researchers have developed a series of safe and effective pest control measures by analyzing the avoidance effects of different stimuli on insect behavior, such as black lights based on phototaxis and trapping devices based on sex pheromones (Lasa et al., 2013). Therefore, it is necessary to research methods for capturing the behavioral trajectories of *T. immissaria*, establishing a foundational data platform for subsequent stimulus experiments. By investigating the correlation between its movement patterns and various types and concentrations of stimuli, we aim to identify optimal pest repellents and concentrations, or enhance the effectiveness of pest trapping devices. This research will ultimately lead to more effective pest management strategies.

To address the above problems, this paper takes *T. immissaria* as the research object, combines thermal infrared technology with deep learning, proposes an thermal infrared multi-target 3D trajectory tracking method. A comprehensive evaluation and comparison of this method have been conducted to address practical problems. This method will help fill the research gap in analyzing nocturnal insect behavior in agriculture and provide observable and quantifiable scientific evidence for precise agricultural pest control.

## 2 Materials and methods

### 2.1 Overview of the *T. immissaria* 3D trajectory acquisition method

**Figure 1:**
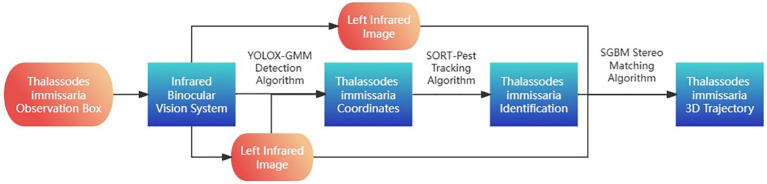
3D trajectory acquisition method for *T. immissaria*.

**Figure 1** shows the overall idea of the *T. immissaria* 3D trajectory acquisition method. First, a video capture device and visual system were designed based on the thermal infrared imaging characteristics. A thermal infrared camera was used to capture images of background modeling in the observation box, and the images were preprocessed to form the Pest_IR dataset. Second, a three-step model for *T. immissaria* 3D trajectory acquisition was constructed. In the first step, all *T. immissaria* targets in the infrared images were detected. In the second step, the detected targets were associated with obtaining the two-dimensional trajectories of each *T. immissaria* strain. In the third step, the 2D trajectories of the *T. immissaria* were matched in stereo to obtain their 3D trajectories.

### 2.2 Experimental platform and image acquisition

**Figure 2:**
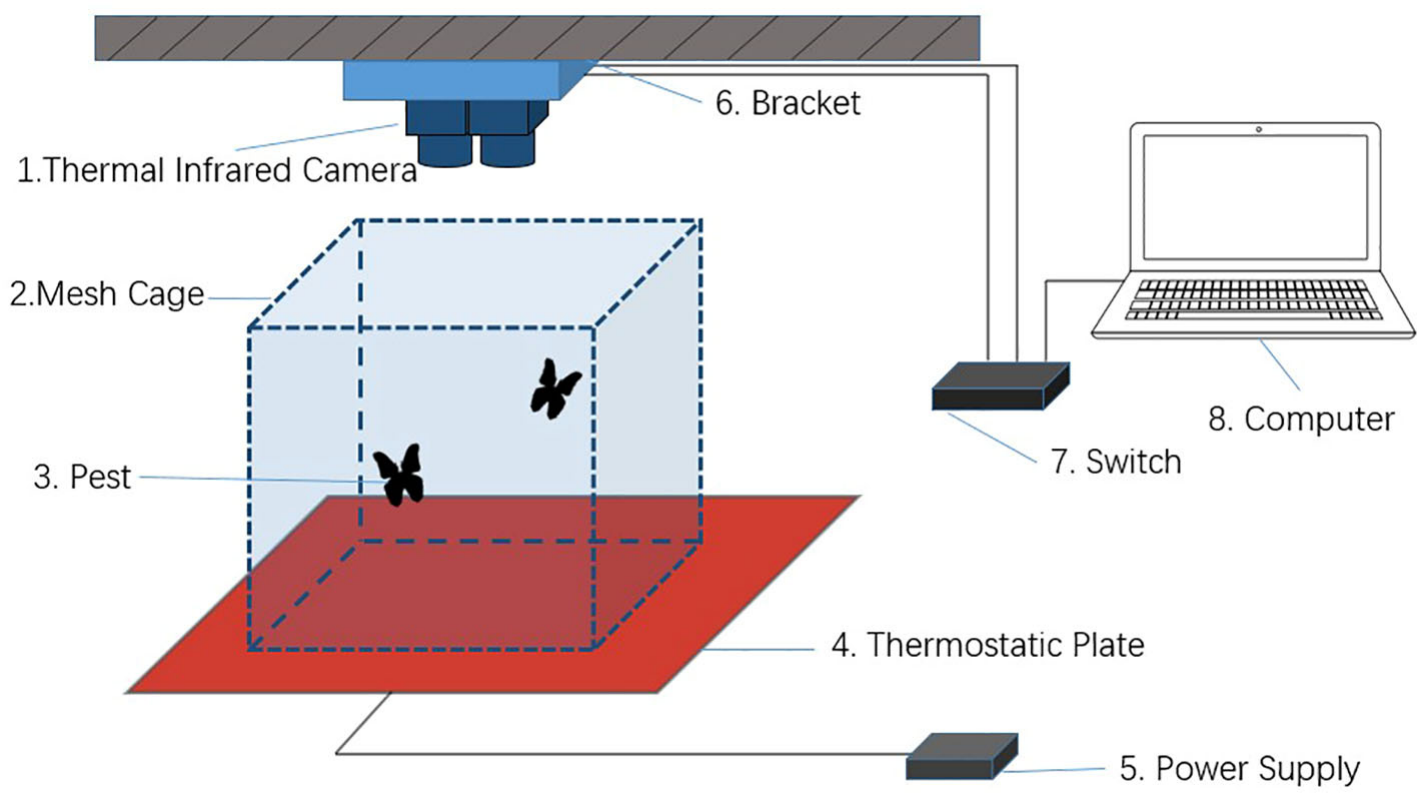
*T. immissaria* infrared video acquisition system.

**Figure 3:**
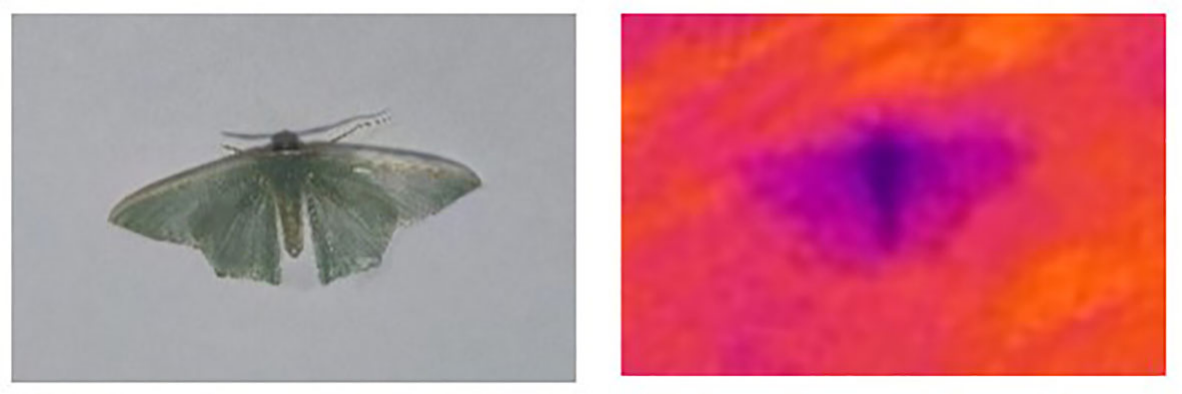
Original image (Left) and thermal infrared image of *T. Immissaria* (Right).

To represent the 3D coordinates of moths in nighttime environments and minimize the influence of human factors, two parallel thermal infrared cameras were used as the image capture system for the moths in this study. The X640D thermal infrared camera produced by Yoseen Company, with a resolution of 640×480 pixels, a frame rate of 30 fps, an FOV of 42×51, and a focal length of 11 mm, were used. These cameras can image objects larger than 0.2 m when the environmental temperature difference exceeds 0.1°C. Considering that *T. immissaria* are holometabolous insects and do not have their own heat characteristics, a constant-temperature heating plate was introduced at the bottom of the observation box. The temperature difference between the geometrids and the heating plate was used as the imaging basis. The heating plate is powered by a 5 V power supply and can maintain a constant temperature of 26°C on the surface, while the indoor temperature is kept constant at 24°C, which is within the optimal range for the growth of *T. immissaria* and does not alter its living environment (Xu et al., 2023). The experiment used a Lenovo laptop connected to the image acquisition system via a switch platform to control the synchronous collection and video data storage. A schematic diagram of the experimental platform and video acquisition is shown in **Figure 2**. The captured thermal infrared images and original images of *T. immissaria* are shown in **Figure 3**.

For better display of image results, this study conducted multiple data collection experiments using a total of 42 *T. immissaria* provided by the Plant Protection Institute of Guangdong Academy of Agricultural Sciences. The eggs of *T. immissaria* were sourced from lychee orchards, and the larvae were fed on tender shoots of lychee trees, cultured in a constant temperature chamber at 26 ± 1 °C with relative humidity maintained between 65% and 85%. The adult *T. immissaria* used in data collection were approximately 28 days old, unmated, and in good health. Each experiment maintained 4-5 *T. immissaria* in a 30×30×30 cm rearing box, fed with a 10% honey solution. The collection time was set from midnight to 6 am, ensuring no bright light throughout the entire period to ensure the diversity of *T. immissaria* movement in the dataset. Finally, a total of 34 segments totaling 14 hours of *T. immissaria* infrared videos were collected in the laboratory environment from September 16, 2023, to October 17, 2023 to construct the dataset.

### 2.3 Dataset preparation

In this study, a dataset was prepared for the deep learning-based object detection model. First, video segments of strong *T. immissaria* activity were manually selected from the images captured by the thermal infrared camera.

**Figure 4:**
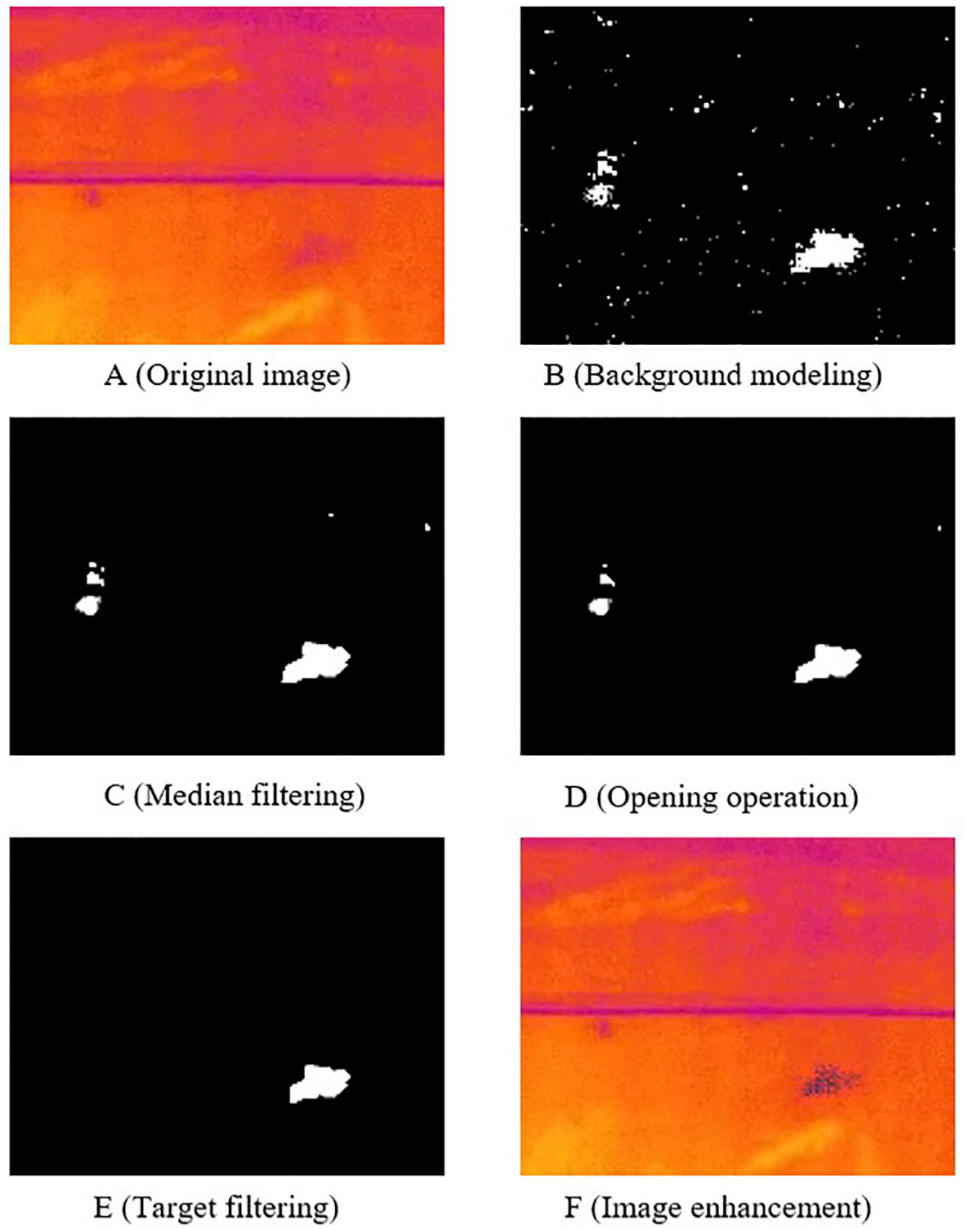
Motion Target Image Enhancement Process. **(A)** Original image. **(B)** Images processed after background modeling. **(C)** Images processed after median filtering. **(D)** Images processed after opening operation. **(E)** Images processed after area filtering. **(F)** Images processed after image enhancement.

For *T. immissaria* in motion, motion blur often occurs due to fast movement, requiring contrast enhancement for blurred targets. The GMM algorithm, as a commonly used motion target detection algorithm in image processing, can segment areas where pixels change by modelling the background (Hu and Zheng, 2016; Liu et al., 2020). After denoising and morphological opening operations on these areas, regions within a specified size range S are selected as motion targets. By overlaying the target on the red channel, the contrast of the target in the thermal infrared image is enhanced, resulting in a clear image of the *T. immissaria*. The image processing workflow is shown in **Figure 4**. By mixing the enhanced images and the original images at a 1:1 ratio, a total of 884 thermal infrared images were obtained. Using the LabelImg image labelling tool, each boundary box of *T. immissaria* in each image was manually labelled to create the Pest_IR dataset for the object detection model, which was then divided into training, validation, and testing sets at an 8:1:1 ratio.

Additionally, to validate the effectiveness of the tracking model, a multitarget tracking dataset was prepared in this study. First, approximately 10 segments of approximately 10 seconds of *T. immissaria* thermal infrared videos were extracted, and the positions of *T. immissaria* in each frame were manually annotated using dark-label video annotation software. These annotations were then converted into the MOT16 standard multitarget tracking dataset MOT_Pest, which includes information such as video frame number, target ID, and target area position.

## 3 Construction of a multiobject 3D trajectory tracking model for *T. immissaria*

In this section, based on the video image data of *T. immissaria* collected by a thermal infrared binocular vision system, a multiobject 3D trajectory tracking scheme for *T. immissaria* is proposed. First, according to the motion characteristics of *T. immissaria*, the YOLOX-GMM object detection algorithm is used to obtain the coordinate information of each *T. immissaria* in the video frames accurately. Then, based on the actual environment inside the observation box, the SORT-Pest algorithm is used to track multiple target outputs by the detection model, and the tracking results for each *T. immissaria* sample are obtained. Finally, based on the 2D tracking results of *T. immissaria* and the pose relationships and intrinsic parameters between the infrared cameras, the semiglobal block matching (SGBM) algorithm is used to reconstruct the 3D trajectory of *T. immissaria*.

### 3.1 YOLOX-GMM object detection algorithm

When *T. immissaria* is stationary or moving slowly, appearance-based object detection algorithms can learn the features of *T. immissaria* well due to the simple image background. Compared with the commonly used YOLO series of object detection algorithms (Redmon and Farhadi, 2017, 2018; Bochkovskiy et al., 2020), the YOLOX algorithm adopts decoupled heads, an anchor-free design, dynamic matching of positive samples, etc., greatly improving the accuracy and speed of the object detection algorithm, which is highly suitable for detecting small targets in thermal infrared images. Therefore, this paper chooses the YOLOX algorithm as the appearance feature detection model for *T. immissaria*.

**Figure 5:**
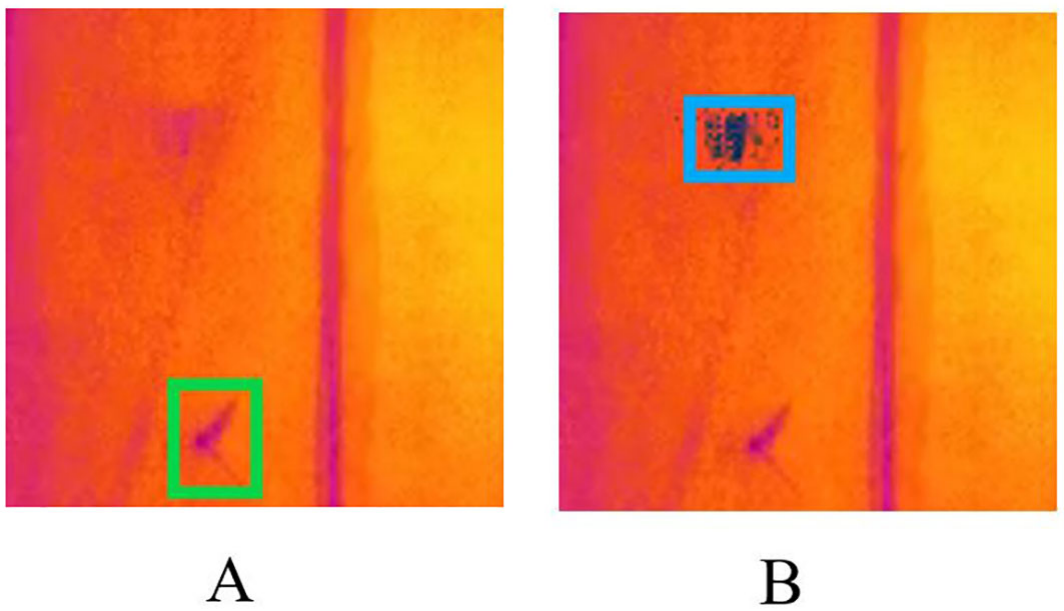
Detection results of *T. immissaria* in different states (**A** shows the detection results of static targets by the YOLOX algorithm, **B** shows the detection results of moving targets by the GMM algorithm).

However, when *T. immissaria* moves at a faster speed, due to the limitation of the video frame rate, the image of *T. immissaria* will exhibit motion blur, which affects the detection accuracy of the appearance feature model. To solve this problem, the GMM algorithm is chosen as the motion feature detection model to detect moving targets. **Figure 5** shows the detection results of the YOLOX algorithm and the GMM algorithm for *T. immissaria* with different motion states in the same frame.

**Figure 6:**
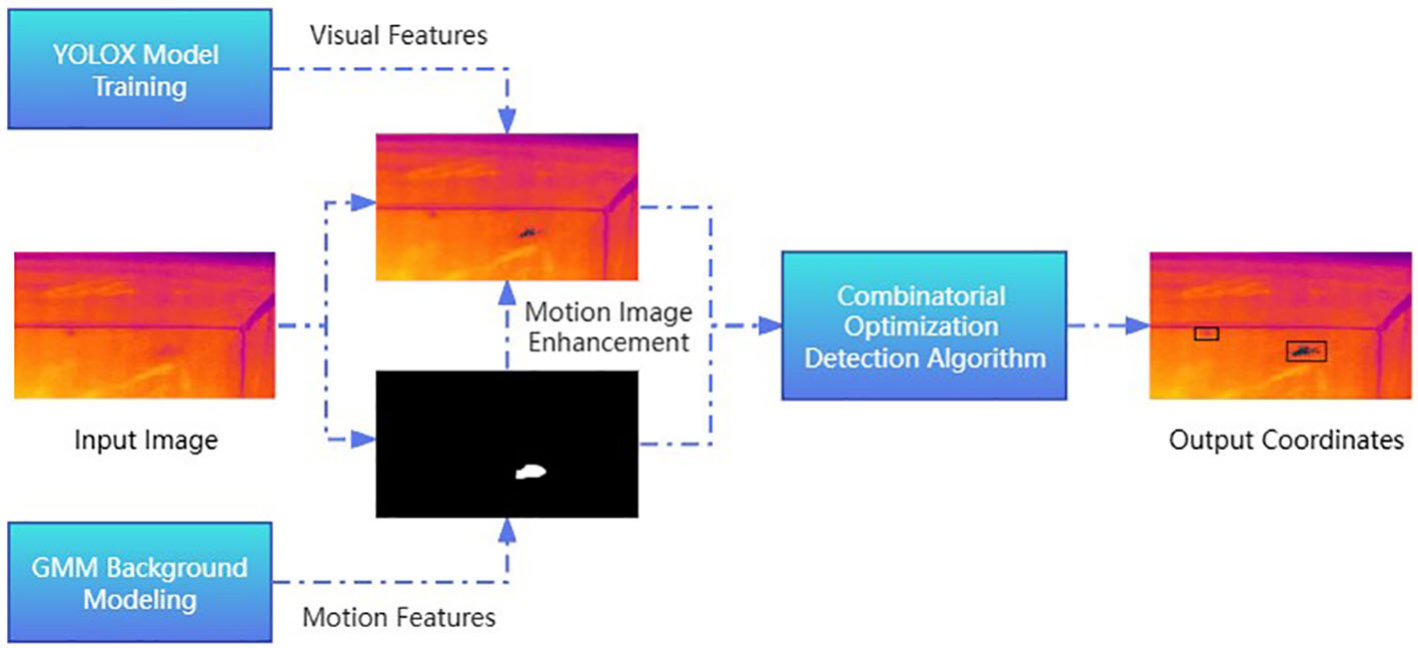
YOLOX-GMM algorithm logic.

The YOLOX-GMM object detection algorithm, which comprehensively considers the appearance and motion features of *T. immissaria* and compares and corrects the detected results, is proposed to accurately detect the entire movement process of *T. immissaria*. The overall logic of the algorithm is shown in **Figure 6**.

First, the GMM algorithm is used to extract i moving targets from each frame image, obtaining their coordinate boxes list1 (x_i_, y_i_, w_i_, h_i_). The pixel size range S of *T. immissaria* is determined to exclude the influence of background factors, and possible moving targets are selected according to the size of S. The image channels corresponding to the original image position are overlaid frame by frame to enhance the contrast of the moving target in the thermal infrared image. Then, the confidence threshold of the YOLOX detector is set to 0.3, and the coordinates of k *T. immissaria* are outputted, forming list 2 (x_k_, y_k_, w_k_, h_k_), to prevent missed detections. The F(x) function is used to compare and correct the output results of list1 from the GMM algorithm and list 2 from the YOLOX algorithm to increase detection result storage and prevent false detections. The correction logic is as follows:

1. If a target is only detected by the YOLOX algorithm, its confidence level is judged. If the confidence level is greater than 0.7, the target is determined to be a true target.
2. If a target is only detected by the GMM algorithm, its consecutive frame appearance count is judged. If the count is greater than 30 frames, the target is determined to be a true target.
3. If a target is detected by both algorithms, the center point positions of the target boxes of the two algorithms are first checked to determine whether they are within a range of 20 pixels. If so, it is determined to be a possible true target and further checked. The intersection-over-union (IOU) ratio of the target boxes of the two algorithms is calculated using Equation 1. If the ratio is greater than 0.5, the target is determined to be a true target.

(1) IOU=Area of Intersection/Area of Union

### 3.2 SORT-Pest object tracking algorithm

**Figure 7:**
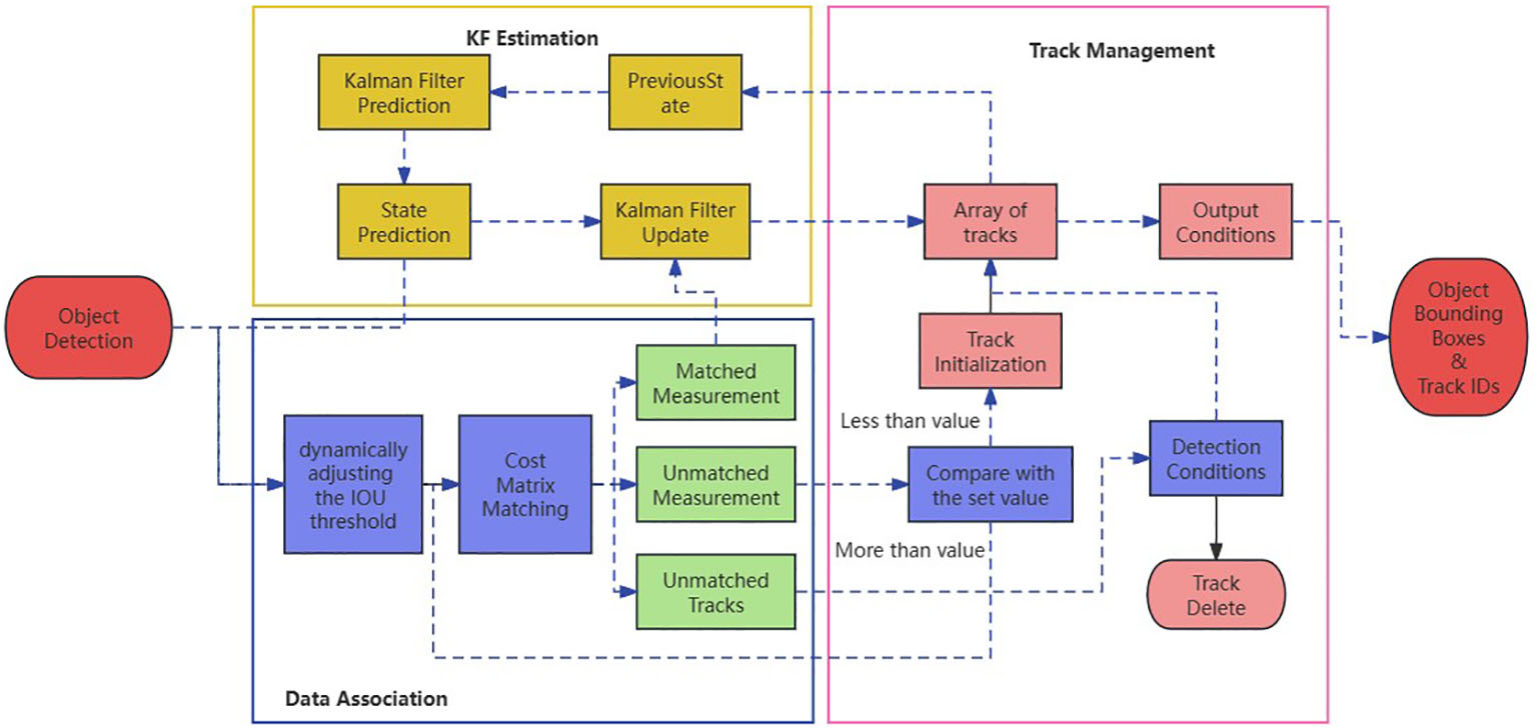
Logic of the SORT-Pest algorithm.

After detecting *T. immissaria*, it is necessary to track it and obtain the 2D trajectory of the entire video sequence. The commonly used multiobject tracking algorithm is the SORT series algorithm (Bewley et al., 2016). Based on the current experimental environment, *T. immissaria* is prone to sudden changes in motion direction and speed within the observation box, which can cause problems such as loss and ID switching in traditional SORT algorithms for multiobject tracking of *T. immissaria*. To solve these issues, this paper proposes two strategies, namely, dynamic IOU matching and cascaded improvement, based on the SORT algorithm, which is called the SORT-Pest algorithm. The overall logic of the algorithm is shown in **Figure 7**.

#### 3.2.1 Dynamic IOU matching

**Figure 8:**
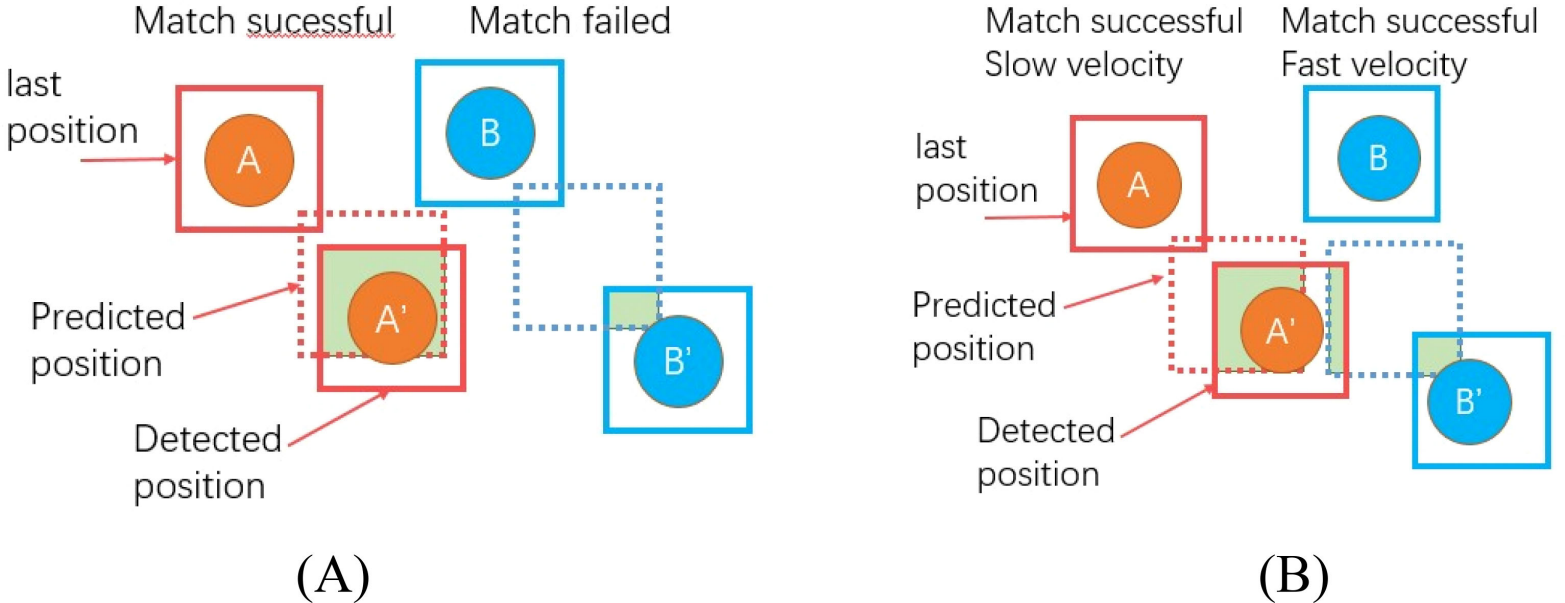
Matching strategy comparison (the green area represents the IOU). **(A)** Associating targets based on a fixed IOU. **(B)** Associating targets by dynamically adjusting the IOU based on velocity.

The SORT algorithm associates targets based on the IOU between the predicted boxes and detected boxes. When the IOU is above a set threshold, it is considered the same target, and when it is below the threshold, it is considered a different target, as shown in **Figure 8A**. However, for fast-moving targets such as *T. immissaria* with significant shape changes, it is difficult for appearance-based reidentification models to associate objects with appearance variations. Moreover, tracking models based on fixed IOU threshold matching are prone to missed detections. Therefore, this paper proposes a strategy for dynamically adjusting the IOU threshold based on the velocity change in *T. immissaria* in each frame. When the velocity is high, the IOU threshold is lowered to tolerate larger position deviations, and when the velocity is slow, the IOU threshold is increased to improve matching accuracy, as shown in **Figure 8B**. This strategy effectively addresses challenges such as target velocity changes and shape variations, improving the accuracy and stability of object tracking.

#### 3.2.2 Cascaded improvement

The SORT algorithm calculates the cost matrix between the detection boxes and trackers and matches them using the Hungarian algorithm. When the matching between the detection box and tracker fails, a tracker is assigned to represent a newly discovered target. However, for enclosed spaces such as observation boxes, the number of targets is fixed. Therefore, this paper proposes an improved cascaded strategy to limit the generation of trackers. After performing the Hungarian algorithm matching, a logical function G(x) is introduced to determine the number of detection boxes. ① When the number of detection boxes is greater than the actual value, it indicates the presence of false alarm targets, and no new trackers are added for unmatched detection boxes. ② When the number of detection boxes equals the actual value, all the targets have been detected. A second matching is conducted for the unmatched detection boxes and trackers, the optimal solution is found, and an ID is assigned based on the Euclidean distance. ③ When the number of detection boxes is less than the actual value, it indicates that there are missed targets. Unmatched trackers are retained for 20 frames until the target reappears. This cascaded approach effectively controls the number of trackers and improves algorithm stability.

### 3.3 3D trajectory reconstruction algorithm

After obtaining the planar trajectory of *T. immissaria*, to reconstruct its 3D trajectory, it is necessary to first match the target images captured by the binocular camera and then combine the internal and external parameters obtained from camera calibration to calculate the disparity map for each frame and convert it into depth information. According to the spatial coordinate transformation relationship of the camera, the correspondence between disparity and depth is shown in Equation 2, and the 3D position relationship of the target relative to the camera is shown in Equation 3.


(2) $$Z_{c}=f*B/d$$

(3) $$P_{c}={\left[X_{c},Y_{c},Z_{c},1\right]}^{T}=inv\left(K\right)*{\left[u,v,1\right]}^{T}*Z_{c}$$

In these formulas, *f* represents the camera focal length, *B* represents the baseline length, *d* represents the left-right image disparity, $P_{c}$ represents the spatial coordinates of the target relative to the camera coordinate system, and *K* represents the camera intrinsic matrix.

The SGBM algorithm is a matching algorithm used to calculate depth information in stereo vision. The main steps are as follows: ① Construct a cost function by measuring the differences between each pixel and other pixels to quantify their relationships. ② Perform cost aggregation by calculating the minimum cost for each pixel using a global path approach. ③ Select the disparity with the minimum accumulated cost as the depth value for each pixel and optimize the final disparity map.

The SGBM algorithm provides good accuracy in-depth information while maintaining relatively high computational efficiency. Therefore, in this paper, the SGBM algorithm is chosen for feature matching and 3D reconstruction of the target tracking results.

## 4 Model experiment and results analysis

### 4.1 Model training and parameter design

This experiment combined the GMM algorithm and YOLOX model to recognize moths. The training and testing processes were conducted on a server running Ubuntu 20.04 LTS. The main hardware devices used were as follows: GPU - RTX A6000, CPU - Intel Core i9-10980XE, and RAM - 64 GB. PyTorch 2.0.1 was utilized as the deep learning framework, and the CNN model was built using the Python programming language. The CUDA version is 11.7, and GPU acceleration is performed using the CUDNN version v8.9.0. The parameters of the YOLOX model were adjusted accordingly to adapt to the specific detection environment of the moths. The input image size of the YOLOX model was set to 640×640 pixels. The IOU threshold was set to 0.5, and a cosine annealing decay strategy was employed to dynamically adjust the learning rate, with a minimum learning rate of 1e-4. The dataset was split into an 80% training set and a 20% validation set. A total of 300 iterations of training were performed, resulting in a series of model parameters that accurately fit the detection boxes of the target moths.

**Figure 9:**
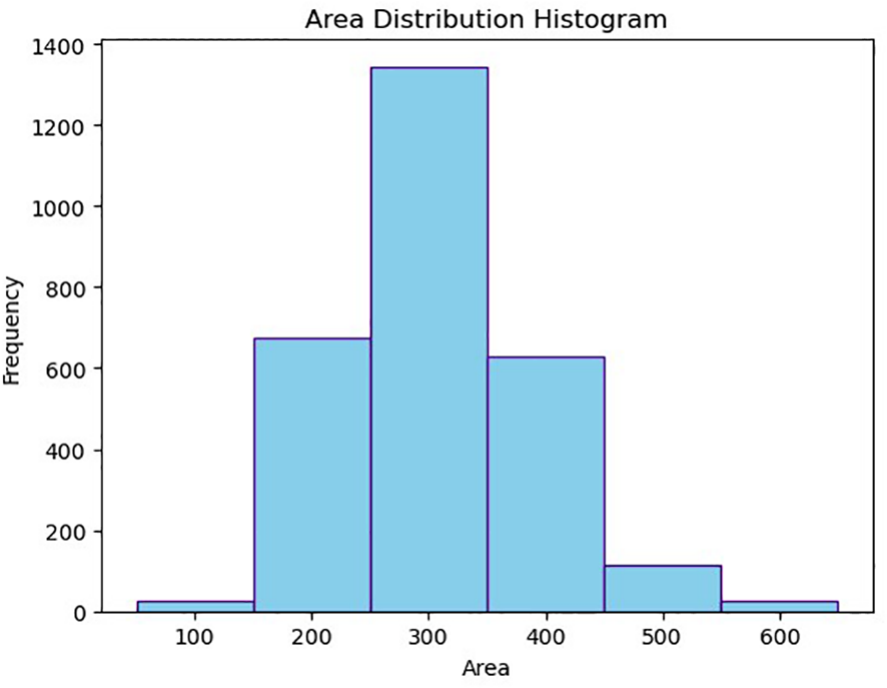
Histogram of the size distribution of *T. immissaria*.

The coordinate information of *T. immissaria* was extracted from the Pest_IR dataset, and its pixel size S was calculated. After removing outliers, the distribution of S is shown in **Figure 9**. It can be observed that S mainly falls within the range of 150-550. This range is used as the threshold for the GMM algorithm to extract moving objects.

Regarding the relationship between the dynamic IOU and velocity in the tracking algorithm, by testing the tracking performance with different threshold values, the optimal dynamic threshold expression is obtained as Equation 4. Based on the average motion speed of *T. immissaria*, the possible Euclidean distance of its motion is calculated as d = 20 pixels, which is used as the threshold for the second matching.


(4) IOU=−0.025v+0.3

### 4.2 Model evaluation metrics

#### 4.2.1 Evaluation indices for the object detection algorithm

In this experiment, R (recall), P (precision), F1 score, and AP (average precision) are used as evaluation metrics for the object detection stage to measure the performance of the model. The formulas for calculating these evaluation metrics are as follows:


(5) $$\text{P}=\frac{TP}{TP+FP}$$

(6) $$\text{R}=\frac{TP}{TP+FN}$$

(7) $$\text{F}1\text{ score}=\frac{2*P*R}{P+R}$$

(8) $$AP=\int_{0}^{1}P\left(R\right)d_{R}$$

where TP represents the number of true positive samples correctly predicted as positive, FN represents the number of true positive samples incorrectly predicted as negative, and FP represents the number of negative samples incorrectly predicted as positive.

#### 4.2.2 Evaluation indices for the tracking algorithm

In this study, the identity switch (IDS), multiple object tracking accuracy (MOTA), and multiple object tracking precision (MOTP) are selected to evaluate the effectiveness of the multiobject tracking algorithm. The IDS represents the number of times the target ID is changed. A smaller value indicates better tracking stability. MOTA refers to the accuracy in tracking multiple targets, including aspects such as missed detections, false alarms, and tracking errors. A higher value indicates better algorithm performance. MOTP refers to the average tracking error of the object’s center point. A smaller value indicates higher detector accuracy. The calculation formulas are as follows:


(9) $$\text{MOTP}=\frac{\sum_{i,t}d_{t}^{i}}{\sum_{t}c_{t}}$$

(10) $$\text{MOTA}=1-\sum_{t}\left(FN_{t}+FP_{t}+IDS_{t}\right)/\sum_{t}GT_{t}$$

where $c_{t}$ represents the number of matches between the detected and predicted targets in frame t, $d_{t}^{i}$ represents the Euclidean distance between the detected and predicted targets in frame t, GT represents the number of true target boxes, and t represents the frame in the video stream.

### 4.3 Results and discussion

#### 4.3.1 The detection model

**Figure 10:**
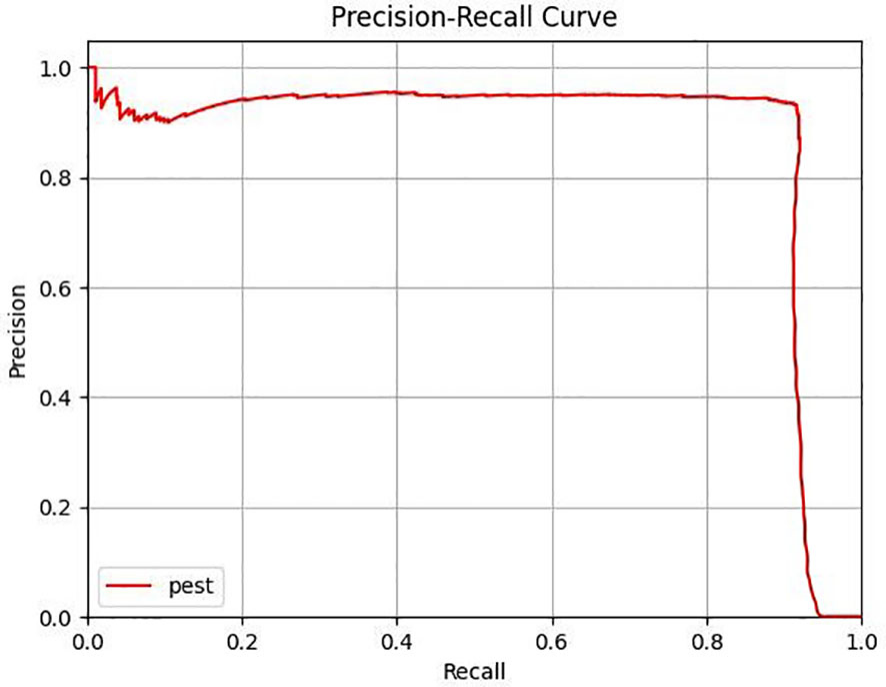
P-R curves of YOLOX detection methods.

To comprehensively evaluate the performance of the YOLOX-GMM model in detecting *T. immissaria*, first, the YOLOX model is trained based on the training parameters set in Section 4.1, and the weight file with the best training effect is used as the test model performance weight file. **Figure 10** shows the P-R curve of the YOLOX model, with the overall P value remaining above 90%. This is because there are many stationary *T. immissaria* in the test set, and the YOLOX algorithm has excellent detection capabilities for static targets, leading to a generally high level of P. Since this paper only focuses on improving moving targets, to highlight the improvement effect of the model, only the evaluation metrics for moving targets are calculated, and the results are shown in **Table 1**. The AP of the YOLOX-GMM model is 89.6%, which is 3.7% higher than that of the original algorithm, mainly reflected in a 5.4% increase in R compared to that of the original algorithm, indicating that the improved model has achieved good detection performance for moving targets.

**Table 1:** Evaluation results of the test dataset for different models.

Models/Evaluation index	AP (%)	R (%)	P (%)	F1 score
YOLOX model	85.9	90.1	97.9	0.938
YOLOX-GMM model	89.6	95.5	99.6	0.975

**Figure 11:**
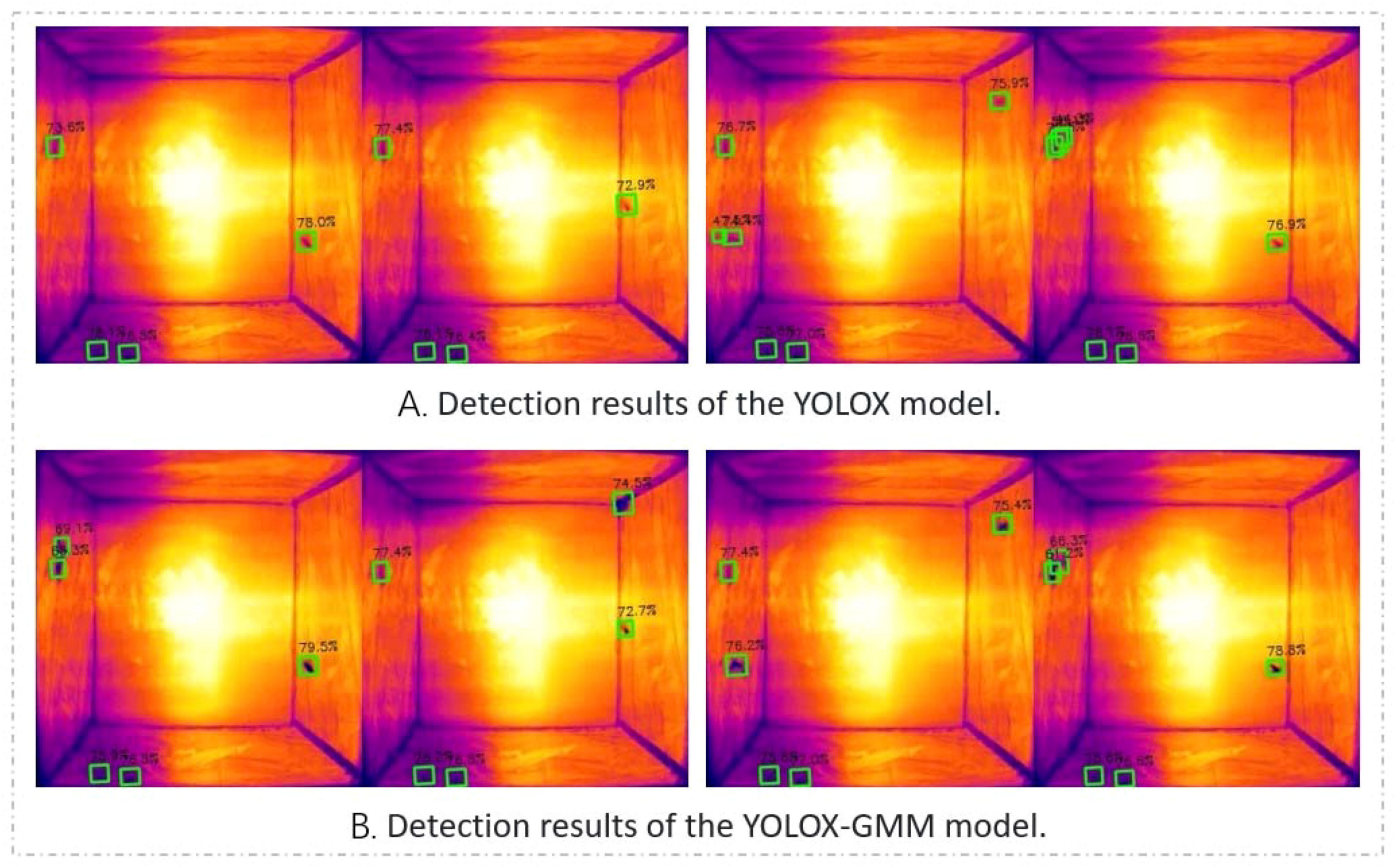
Target detection results (there are actually five *T. immissaria* in the image, the left two columns show the results of the missed detection comparison, and the right two columns show the results of the false alarm comparison). **(A)** Detection results of the YOLOX model. **(B)** Detection results of the YOLOX-GMM model.

**Figure 11** compares the detection effects of the two algorithms on *T. immissaria*. From the detection results, From the detection results, the YOLOX algorithm encounters challenges in detecting images with motion blur, leading to a significant number of missed detections. In contrast, the proposed algorithm effectively restores the clarity of blurred targets and improves target recognition rate. Additionally, when addressing the background noise problem in the infrared image, under the same confidence threshold of 0.3, the YOLOX-GMM algorithm has a lower false alarm rate. Overall, the YOLOX-GMM object detection model adapts well to the experimental environment and is far superior to the original algorithm in terms of the recognition accuracy of moving targets in thermal infrared images. Simultaneously, an analysis was conducted on the images where the YOLOX-GMM algorithm failed to detect targets, revealing that the main reason was the uneven heating at the edge of the constant-temperature plate, resulting in imaging failure of *T. immissaria*. However, this issue can be addressed through subsequent hardware optimizations to enhance the practical detection performance of the algorithm.

#### 4.3.2 The tracking model

To validate the effectiveness of the proposed multiobject tracking algorithm SORT-Pest in this study, we compared different strategies of the SORT algorithm using the validation set of the Pest_IR dataset. To ensure fairness in the experiments, we uniformly used the YOLOX-GMM model as the detector. The experimental results are shown in **Table 2**.

**Table 2:** Evaluation index results obtained by different models.

Models/Evaluation index	IDS	MOTA (%)	MOTP
SORT model	47	75.4	0.43
Sort model + IOU	27	87.6	0.44
Sort model + Cascade	7	91.7	0.45
SORT-Pest model	3	96.9	0.45

**Figure 12:**
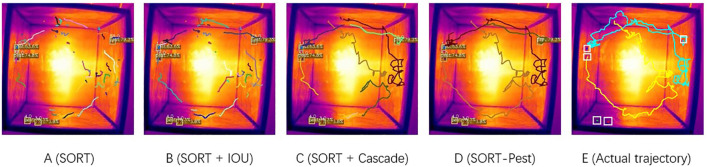
Trajectory tracking results under different strategies (colors of trajectories represent the number of trackers). **(A)** Tracking results of the original SORT model. **(B)** Tracking results of the SORT + IOU matching model. **(C)** Tracking results of the SORT + Cascaded improvement model. **(D)** Tracking results of the SORT-Pest model. **(E)** Actual motion trajectories of *T. immissaria*.

A comparison between the actual trajectories using four tracking models to test the effectiveness on a 276-frame video is shown in **Figure 12**. The experimental results indicate that the improvement strategy proposed for the traditional SORT algorithm effectively addresses the issues of ID switching and tracking loss. This is because the SORT-Pest model accounts for the fixed quantity of *T. immissaria* in the observation box, thus strictly limiting the generation of trackers. For the first unmatched detection box and tracker, distance threshold matching is performed again, reducing the frequency of ID switching. Furthermore, considering the change in the speed of *T. immissaria* during the tracking process, the dynamic IOU matching strategy can appropriately adjust the threshold according to the speed of the tracker when the speed of *T. immissaria* changes suddenly, thereby improving the stability of the model’s tracking.

#### 4.3.3 The 3D trajectory reconstruction model

##### 4.3.3.1 Tracking accuracy comparison

**Figure 13:**
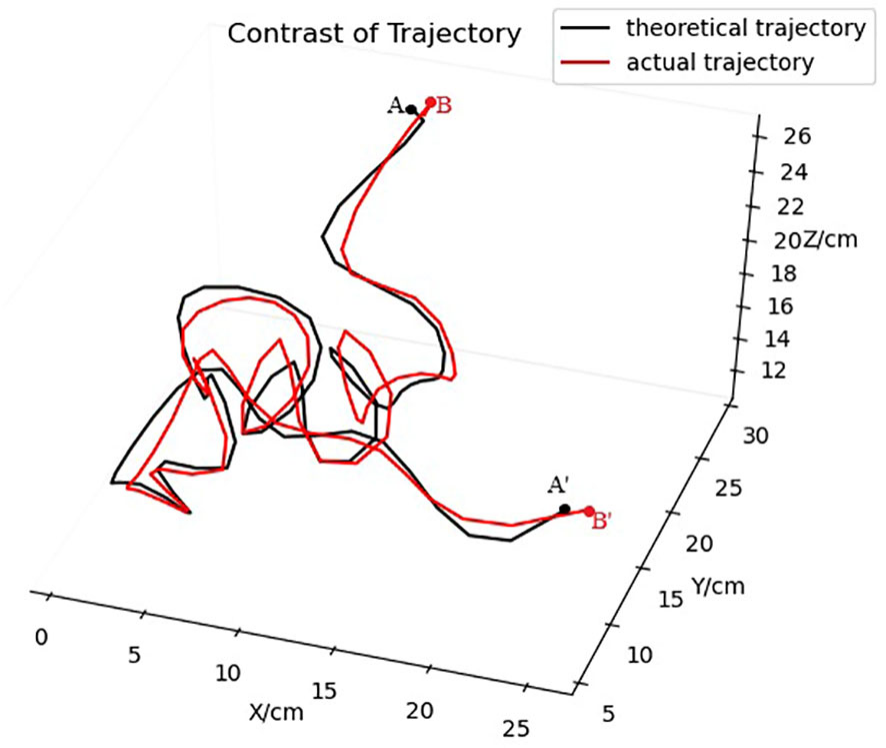
3D trajectory comparison (the actual trajectory is drawn based on the annotation results, while the theoretical trajectory is drawn based on the tracking matching results). Points A and B represent the starting points, and points A’ and B’ represent the endpoints).

Based on the stereo matching results of the SGBM algorithm on the infrared images from the left and right cameras and considering the pose relationship between the cameras, the output trajectories of the tracking model were reconstructed in 3D to obtain the 3D spatial coordinates of the *T. immissaria* for each frame. The 3D trajectory of *T. immissaria* was plotted using MATLAB software and then compared with the true 3D trajectory drawn based on the annotation results, as shown in **Figure 13**. The comparison result indicates a high degree of overlap between the two trajectories, thereby validating the accuracy of the tracking algorithm proposed in this study for tracing the trajectory of *T. immissaria*.

##### 4.3.3.2 Evaluation of 3D coordinate accuracy

Because it is difficult to obtain the true spatial coordinates of *T. immissaria* in the actual environment, this study chose LED lights similar in size to *T. immissaria* to evaluate the accuracy of the proposed method in determining the 3D coordinates of insect targets. Within the observation box, 10 LED lights were hung at different positions using thin wires, and the actual 3D spatial coordinates of each LED light were measured using a tape measure. By applying the method described in this study, the theoretical 3D spatial coordinates of each LED light were obtained. The 3D spatial coordinates of the 10 LED lights were obtained using both methods and then transformed into a coordinate system with the camera center as the origin, as shown in **Table 3**. The minimum error calculated for these points was 8 mm, the maximum error was 19 mm, the average error was 15 mm, and the standard deviation was 3.8 mm. The average error range is close to the size of *T. immissaria*, demonstrating that it essentially meets the accuracy requirements of the 3D coordinates of *T.immissaria*.

**Table 3:** Comparison of 3D coordinate results.

Number	Theoretical coordinates (cm)	Actual coordinates (cm)	Error (cm)
1	(7.7, -8.1, 43.4)	(7.7, -8.2, 45.3)	1.90
2	(-10.9, 0.2, 32.67)	(-9.7, 0.7, 32.0)	1.46
3	(-14.7, -12.6, 42.7)	(-15.1, -11.4, 43.2)	1.36
4	(-12.4, 5.0, 46.0)	(-11.8, 5.4, 46.3)	0.78
5	(-5.7, 7.2, 34.1)	(-4.8, 8.1, 35.4)	1.82
6	(8.5, 12.8, 32.3)	(9.9, 11.6, 32.1)	1.85
7	(-3.0, 3.2, 48.8)	(-2.3, 2.4, 50.0)	1.60
8	(12.6, -8.0, 37.2)	(11.2, -8.6, 36.4)	1.72
9	(-13.4, 6.6, 37.5)	(-13.2, 5.5, 36.9)	1.27
10	(2.5, 4.8, 48.9)	(1.9, 5.1, 49.6)	0.97

#### 4.3.4 Innovations and limitations

Currently, behavioral analysis of insects is primarily conducted using RGB cameras, achieving notable success in species detection, behavior recognition, and trajectory tracking. However, for light-sensitive nocturnal insects, the accuracy of behavioral analysis using RGB cameras is often compromised due to limitations in light sources. Therefore, this study innovatively introduces thermal infrared cameras as imaging devices, enabling trajectory tracking of nocturnal insects without the need for lighting and providing more authentic behavioral data.

In contrast to RGB cameras, which are highly sensitive to visual features and require separate deep learning models even for similar insects of the same genus, thermal infrared imaging technology effectively mitigates the visual distinctiveness of insects in incubation chambers. This allows tracking of multiple congeneric insects using a single deep learning model, significantly reducing system application costs. Furthermore, traditional manual observation methods face challenges and errors, particularly with small-bodied and fast-flying insects, often constrained to two-dimensional planes (Machraoui et al., 2019). Although some studies have achieved three-dimensional trajectories of insects, they often lack real-world coordinates and error calculations (Nasir et al., 2021; Chen et al., 2021).

In this study, using binocular cameras, we not only achieved simultaneous three-dimensional positioning of multiple insects but also accurately calculated positioning errors. Although the average error in this study is greater compared to the 8.8mm error achieved by Guo et al. (2018) in 3D spatial positioning of cabbage butterfly using RGB stereo cameras, this research addresses the precise measurement of nocturnal insect positioning in low-light environments, a challenge RGB cameras cannot meet. Furthermore, with the ongoing development of thermal infrared imaging technology, this error is expected to decrease gradually in the future. Consequently, the value of this research in the field of insect behavioral analysis is anticipated to increase.

Nevertheless, due to time and resource constraints, this study remains technically focused, lacking specific behavioral analysis experiments as its major limitation. Future research will focus on exploring the effects of different times, temperatures, and chemical hormones on the movement trajectories of *T. immissaria*, documenting and summarizing their motion data and behavioral patterns. These findings will assist in determining the most suitable repellents and concentrations based on the insects’ responses to various stimuli, or optimizing trap placement angles according to their behavioral patterns, thereby providing substantial information support for refining pest management strategies.

## 5 Conclusion

This study proposed and evaluated a multiobject 3D trajectory tracking method for *T. immissaria* based on thermal infrared video. The proposed method consists of three main parts. Firstly, a YOLOX-GMM combined optimization detection algorithm is proposed by integrating deep learning and image processing techniques to address the challenges of small and difficult-to-detect objects in infrared videos, significantly improving the accuracy of *T. immissaria* recognition. Additionally, this method significantly reduces the missed detection rate and false alarm rate, providing a more reliable solution for small object detection in thermal infrared videos.

In the second step, the detection results are tracked using the proposed SORT-Pest tracking model, which significantly reduces the ID switch frequency and achieves a 96.9% MOTA by introducing dynamic IOU matching and cascade improvement strategies. Compared to traditional multi-object tracking algorithms (such as SORT and DeepSort), the proposed method effectively addresses the difficulties of tracking fast and irregularly moving targets, improving the accuracy and consistency of target tracking.

The third part involves the use of a thermal infrared binocular vision system to reconstruct the motion trajectories of *T. immissaria* in three dimensions. The average positioning error of the reconstructed 3D space is 15 millimeters, which basically meets the accuracy requirements for quantitative behavioral analysis of *T. immissaria*.

Overall, the proposed method can accurately obtain the three-dimensional trajectories of *T. immissaria* and other insects regardless of lighting conditions, providing important innovation and contributions to the development of insect behavior analysis systems and having broad application prospects in the field of plant protection. This study serves as the foundation for the behavioral analysis of nocturnal insects like *T. immissaria.* The next step will focus on how their behavior patterns can contribute substantively to pest management strategies.

## Data Availability

The raw data supporting the conclusions of this article will be made available by the authors, without undue reservation.
